# Supplementary material for: Vitamin D Modified DSS-Induced Colitis in Mice via STING Signaling Pathway
Source: Biology (Basel). 2025 Jun 18;14(6):715. doi: 10.3390/biology14060715 (PMC12190092; doi:10.3390/biology14060715)
Supplement: Supplementary file 1 [file biology-14-00715-s001.zip › Supplementary Materials S1: 16s-report of gut microbiota/Groups/diff_analysis/heatmap/Speciesdiffheatmap.pdf]

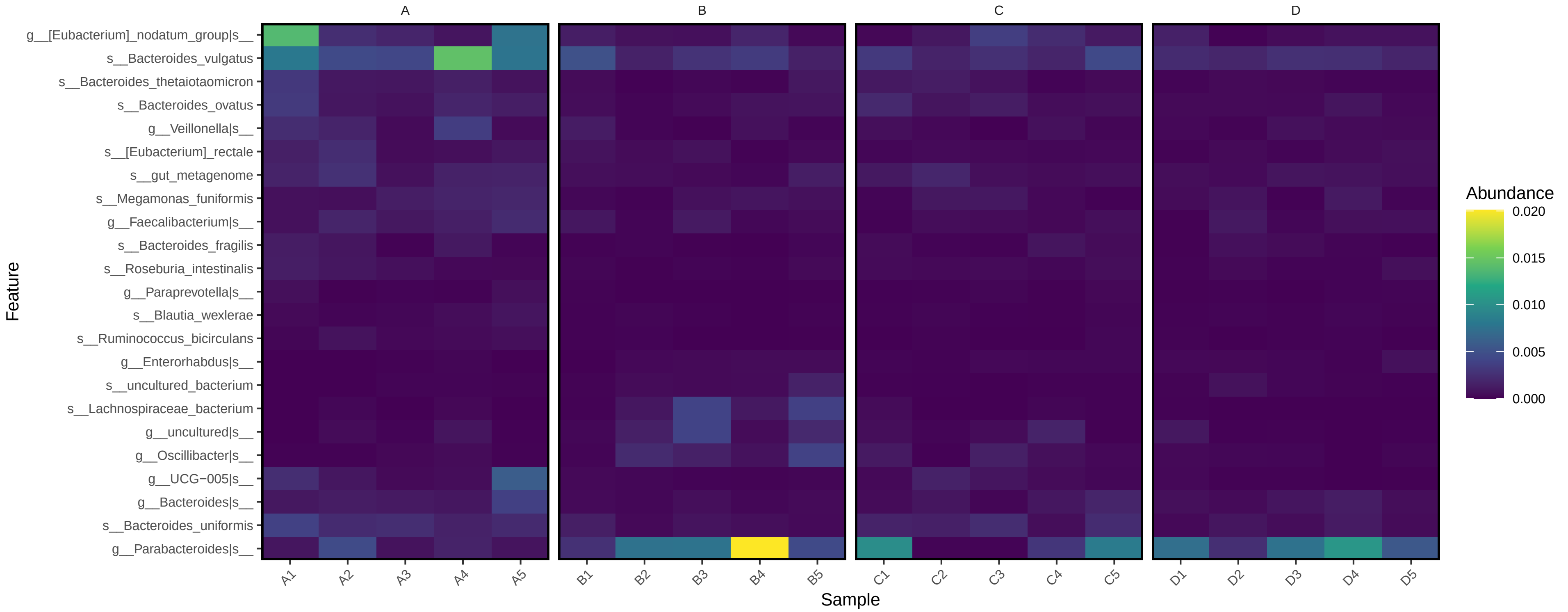

A
B
C
D
Feature
g__[Eubacterium]_nodatum_group|s__
s__Bacteroides_vulgatus
s__Bacteroides_thetaiotaomicron
s__Bacteroides_ovatus
g__Veillonella|s__
s__[Eubacterium]_rectale
s__gut_metagenome
s__Megamonas_funiformis
g__Faecalibacterium|s__
s__Bacteroides_fragilis
s__Roseburia_intestinalis
g__Paraprevotella|s__
s__Blautia_wexlerae
s__Ruminococcus_bicirculans
g__Enterorhabdus|s__
s__uncultured_bacterium
s__Lachnospiraceae_bacterium
g__uncultured|s__
g__Oscillibacter|s__
g__UCG−005|s__
g__Bacteroides|s__
s__Bacteroides_uniformis
g__Parabacteroides|s__
A1
A2
A3
A4
A5
B1
B2
B3
B4
B5
C1
C2
C3
C4
C5
D1
D2
D3
D4
D5
Sample
Abundance
0.020
0.015
0.010
0.005
0.000
